# A Case Report of Laparoscopic Cholecystectomy in an Obese Patient With Situs Inversus Totalis

**DOI:** 10.7759/cureus.104537

**Published:** 2026-03-02

**Authors:** Shohei Maruta, Tetsuro Ohno, Masami Yamada, Chiaki Fukuda, Akira Maki

**Affiliations:** 1 Department of Hepato-Biliary-Pancreatic Surgery and Pediatric Surgery, Saitama Medical Center, Saitama Medical University, Kawagoe, JPN; 2 Department of Surgery, Chichibu Hospital, Chichibu, JPN; 3 Department of Internal Medicine, Chichibu Hospital, Chichibu, JPN

**Keywords:** body mass index (bmi), cholelithiasis, laparoscopic cholecystectomy, obesity, situs inversus totalis

## Abstract

Situs inversus totalis (SIT) is a rare congenital condition characterized by a complete mirror-image arrangement of the visceral organs. We report a laparoscopic cholecystectomy performed on an obese patient with a body mass index (BMI) over 30 kg/m² and SIT. An 80-year-old female patient with SIT and a BMI of 30.1 kg/m² was diagnosed with cholecystolithiasis. Laparoscopic cholecystectomy was performed using a mirror-image port placement relative to the standard configuration. The postoperative course was uneventful. Laparoscopic cholecystectomy can be safely performed in patients with SIT, even in those with obesity, defined as a BMI greater than 30 kg/m².

## Introduction

Situs inversus totalis (SIT) is a rare congenital condition characterized by a complete reversal of the internal organs. The incidence of SIT has been reported to vary widely, ranging from 1:5,000 to 1:20,000 [1]. Surgical procedures in these patients are considered technically challenging due to the mirror-image anatomy and potential associated anatomical variations. The first successful laparoscopic cholecystectomy in a patient with SIT was reported by Campos and Sipes in 1991 [2,3], and subsequent case reports have gradually accumulated. Surgical procedures in obese patients are also technically demanding because of factors such as increased visceral fat and a restricted operative field. Earlier reports suggested that obesity was associated with a higher conversion rate during laparoscopic cholecystectomy, particularly in patients with severe obesity (body mass index (BMI) ≥35-40 kg/m²) or concomitant acute cholecystitis. Nevertheless, with improvements in laparoscopic technology and surgical expertise, more recent data indicate that the impact of class I obesity on conversion rates may be less pronounced, especially in elective cases of uncomplicated cholelithiasis [3]. According to the World Health Organization, obesity is defined as a BMI ≥30 kg/m², with BMI 30.0-34.9 kg/m² classified as class I obesity. The present case fell into this category. Although obesity can increase operative difficulty in some patients due to increased visceral fat or reduced working space, our patient did not demonstrate extreme technical challenges such as difficulty with trocar insertion, pneumoperitoneum establishment, or excessive bleeding. Between 1990 and 2022, the global prevalence of obesity among adults aged 18 years and older more than doubled, increasing from 7% to 16%. As the prevalence of obesity continues to rise, the number of surgical procedures performed on obese patients is expected to increase accordingly. A limited number of cases of laparoscopic cholecystectomy in patients with SIT and obesity have been reported in the literature. A PubMed search using the terms “situs inversus totalis”, “obesity”, and “laparoscopic cholecystectomy” identified one reported case at the time of review. These findings indicate that evidence remains limited, and further case accumulation is warranted to better clarify surgical considerations in this specific subgroup. In this report, we present a case of successful laparoscopic cholecystectomy in a patient with both SIT and obesity and discuss the surgical considerations relevant to this condition.

## Case presentation

**Figure 1:**
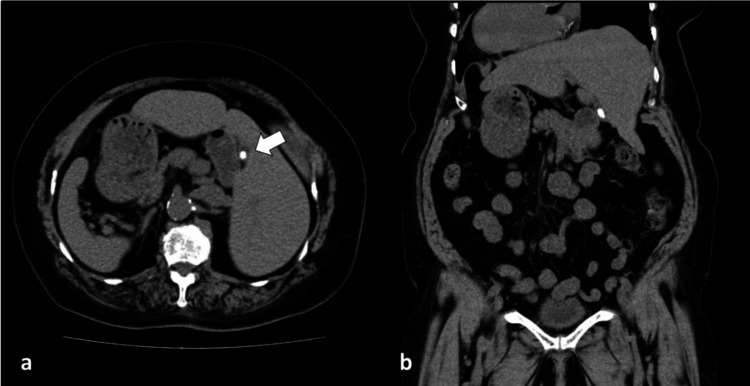
(a) Axial CT images revealing a 15-mm gallstone within the gallbladder (arrow), along with increased adipose tissue consistent with obesity. (b) Coronal CT images showing that all organs are arranged in a mirror-image configuration compared with the normal anatomy, consistent with situs inversus totalis CT: computed tomography

An 80-year-old woman with SIT, with a height of 155 cm and a weight of 75.5 kg, presented with a three-month history of recurrent left upper quadrant pain. She had previously been informed that she had a gallstone and had been diagnosed with SIT. Her BMI at the time of her first visit to our hospital was 30.1 kg/m². On physical examination, her abdomen was soft and nontender. Laboratory tests were within normal limits except for a mildly elevated C-reactive protein (CRP) level of 2.20 mg/dL (Table 1). The patient showed no clinical or imaging findings suggestive of acute cholecystitis, and the procedure was performed electively. The elevated CRP level was, therefore, considered to reflect chronic inflammatory changes associated with chronic cholecystitis. Chest X-ray demonstrated dextrocardia, and plain computed tomography (CT) revealed a mirror-image arrangement of the internal organs, polysplenia, and a solitary 15-mm gallstone (Figure 1). Contrast-enhanced CT was not performed because of the patient’s history of bronchial asthma. Although she had no known contrast allergy, contrast use was avoided as a precaution. Magnetic resonance cholangiopancreatography (MRCP) showed no abnormalities of the biliary tree. Based on these findings, a laparoscopic cholecystectomy was planned for cholelithiasis.

**Table 1:** Laboratory findings ALP: alkaline phosphatase; AST: aspartate transaminase; ALT: alanine transaminase; γ-GTP: gamma-glutamyl transpeptidase; PT: prothrombin time; INR: international normalized ratio; APTT: activated partial thromboplastin time

Parameter	Patient value	Reference range	Unit
Albumin	6.6	6.7-8.3	g/dL
Total bilirubin	0.2	0.2-1.0	mg/dL
ALP	53	38-113	U/L
AST	18	8-38	U/L
ALT	12	4-43	U/L
γ-GTP	15	<48	U/L
Creatinine	0.85	0.47-0.79	mg/dL
Amylase	45	37-125	U/L
C-reactive protein	2.2	<0.30	mg/dL
White blood cell count	6,200	3,500-9,100	/µL
Hemoglobin	11.8	11.3-15.2	g/dL
Platelet count	18.8	13.0-36.9	×10⁴/µL
PT-INR	0.96	0.90-1.10	-
APTT	26.6	25-40	Seconds

**Figure 2:**
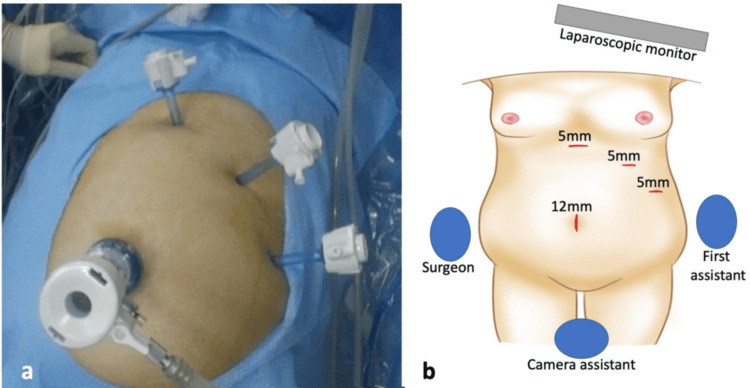
Port positions. (a) Intraoperative view showing ports arranged in a mirror-image configuration. (b) Illustration of the setup and port placement Image credit: Figure 2b is an original image created by the author Shohei Maruta using MediBang Paint

The primary surgeon, who was right-handed, was positioned on the patient’s right side, with the assistant on the left side and the camera operator between the patient’s legs. A 12-mm trocar was introduced at the umbilicus using an open laparoscopic technique. Three additional 5-mm trocars were inserted under direct visualization in the subxiphoid, left subcostal, and left lateral regions (Figure 2). Laparoscopy demonstrated reversed abdominal anatomy and abundant intra-abdominal fat. The surgeon primarily used instruments inserted through the subxiphoid and left subcostal trocars. The gallbladder fundus was grasped via the left lateral trocar and retracted cranially. The gallbladder showed no signs of inflammation and was easily manipulated.

**Figure 3:**
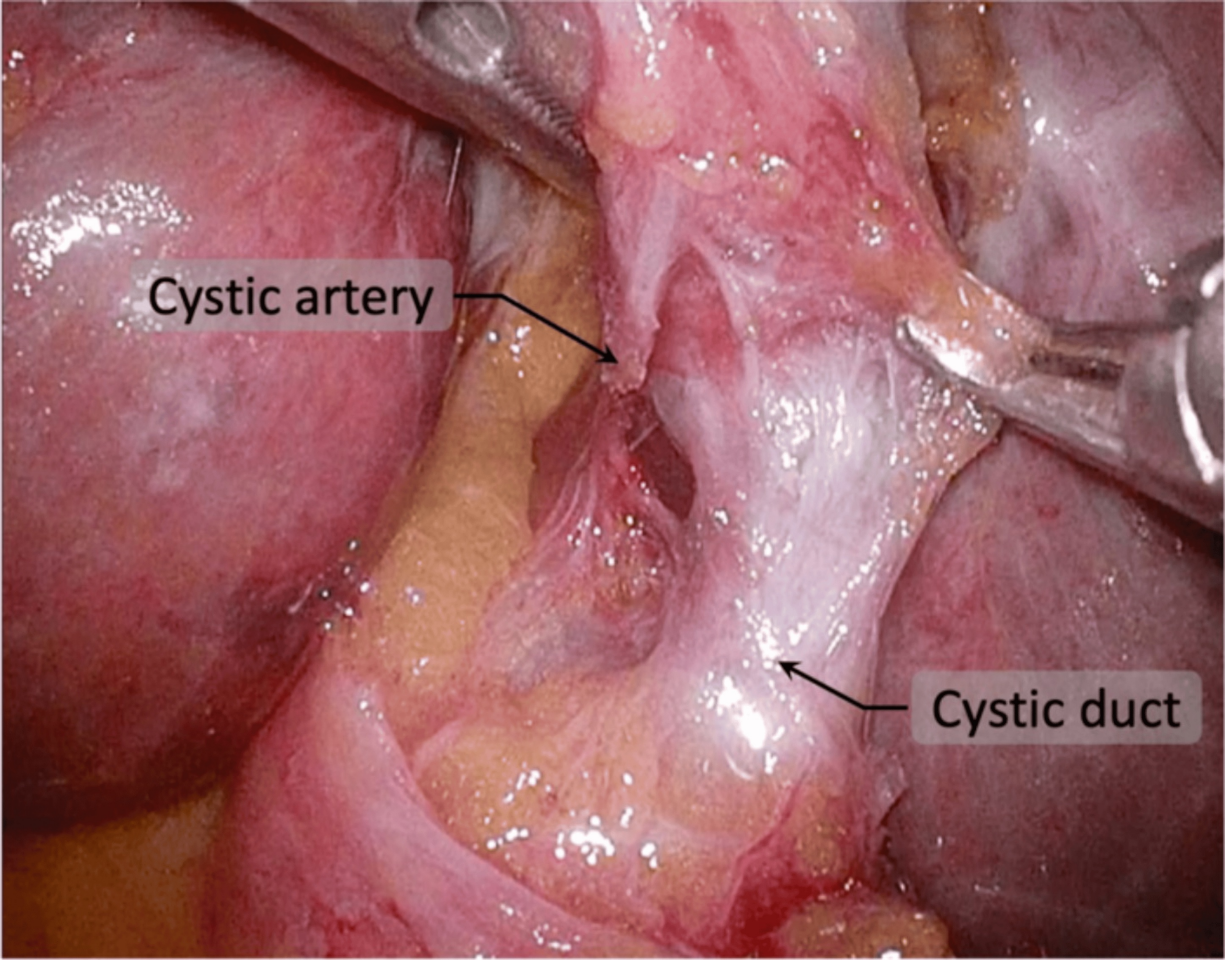
CVS appearing as a mirror image and was clearly confirmed CVS: critical view of safety

**Figure 4:**
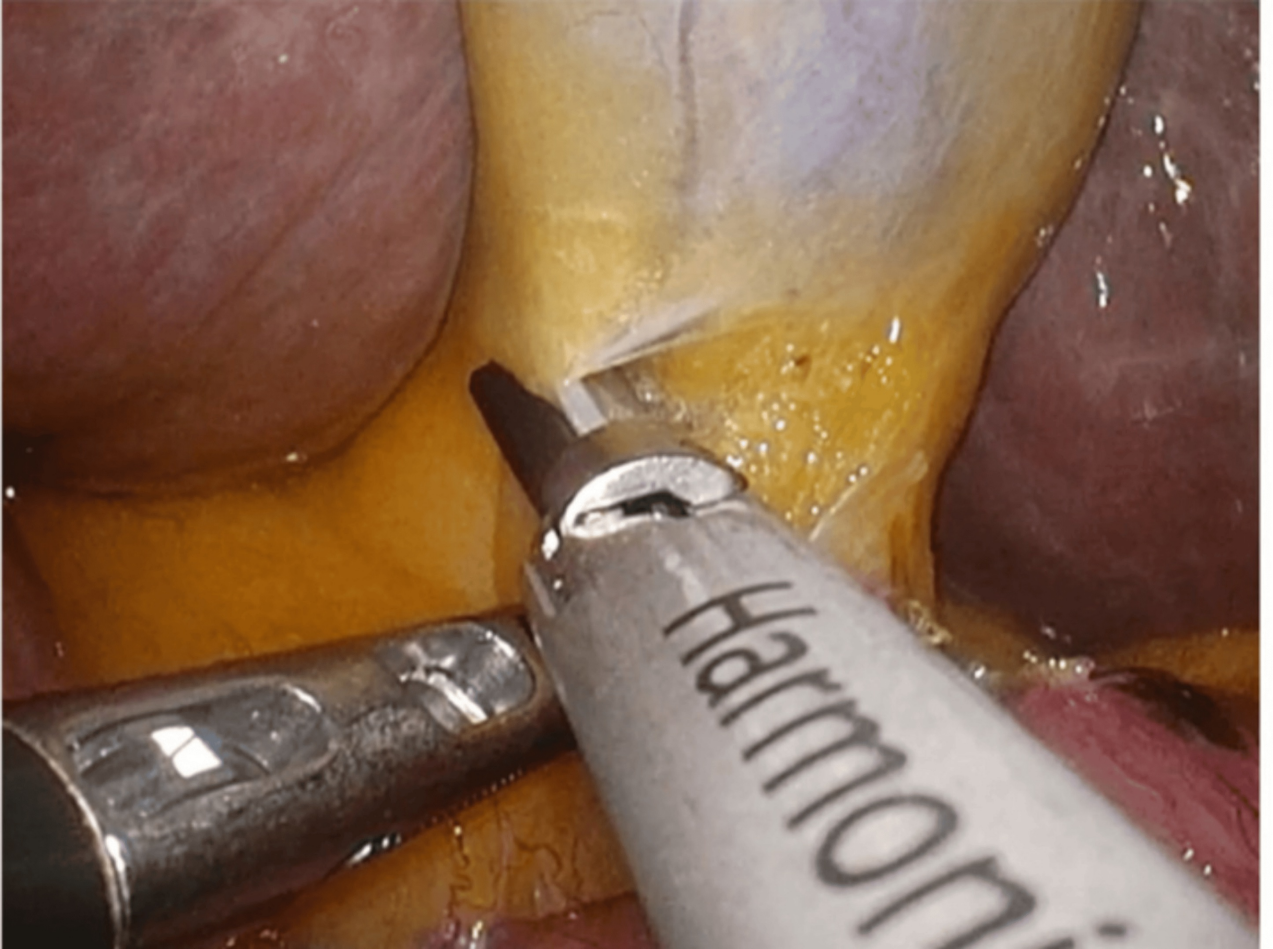
Dissection of the hepatocystic triangle, demonstrating crossing of the left and right forceps during the procedure

The serosa of the gallbladder neck was incised to identify the cystic duct and artery. The hepatocystic triangle was fully cleared of fibrofatty tissue, and the lower third of the gallbladder was dissected from the liver bed to obtain the critical view of safety (CVS) (Figure 3). Only two structures entering the gallbladder were identified before clipping. Intraoperative cholangiography was not performed because the biliary anatomy was clearly visualized. Dissection of the hepatocystic triangle was technically challenging because the right-hand port, typically used for the energy device, was positioned contralateral to the gallbladder. To overcome this, the surgeon intermittently switched dominant and assisting hands during dissection to maintain appropriate traction and visualization. In addition, minor adjustments in instrument handling were made to reduce crossing and improve ergonomics (Figure 4). The camera angle was adjusted as needed, and the use of a 30° laparoscope facilitated visualization of the biliary structures. The cystic artery was sealed and divided using an energy device, and the cystic duct was doubly clipped and divided. The gallbladder was detached from its hepatic bed and extracted through the umbilical wound using a retrieval bag. The operation time was 156 minutes, and blood loss was minimal. At our institution, elective laparoscopic cholecystectomy is typically completed within 60-90 minutes. The prolonged operative time in this case was mainly attributed to the reversed anatomy in SIT, which required careful dissection and intermittent hand switching. Instrument crossing also contributed to the technical difficulty, whereas obesity had a minimal impact on the procedure.

Gross examination of the specimen revealed a single 15-mm black stone, with no mucosal lesions suggestive of malignancy. Histopathological examination revealed lymphocytic infiltration of the mucosa and gallbladder wall, consistent with a diagnosis of chronic cholecystitis. The patient's postoperative course was uneventful, and she was discharged on postoperative day 3.

## Discussion

Situs inversus is an uncommon congenital condition that typically follows an autosomal-recessive inheritance pattern. Its estimated frequency ranges from approximately one in 5,000 to one in 20,000 live births [1]. The term refers to a continuum of visceral transposition. In its complete form (SIT), both thoracic and abdominal organs are reversed, producing a mirror-image arrangement of normal anatomy. In partial forms (situs inversus partialis), the reversal is limited to either the thoracic or the abdominal organs [4]. Kartagener’s syndrome, which was first described by Siewert in 1904 [5] and later defined as a distinct clinical entity by Kartagener in 1933 [6], is an autosomal-recessive genetic disorder characterized by chronic sinusitis, bronchiectasis, and situs inversus. The patient had a history of bronchial asthma but showed no evidence of sinusitis and therefore did not meet the diagnostic criteria for Kartagener’s syndrome.

There are two major reasons why laparoscopic cholecystectomy in patients with situs inversus is challenging. The first relates to anatomical factors, and the second to technical considerations. From an anatomical perspective, patients with complete situs inversus are at increased risk of anatomical misidentification because all organs are positioned as mirror images of their usual locations. Patients with SIT have been reported to exhibit a higher incidence of congenital anatomical anomalies, including cardiovascular, renal, and biliary abnormalities [7]. Vascular variations, such as anomalies of the celiac trunk and hepatic arteries, may also be present. A duplicated cystic artery has also been reported in patients with SIT [8], which may increase technical complexity during surgery. Given these considerations, it is advisable to conduct a thorough preoperative imaging evaluation to identify any anatomical variations as comprehensively as possible in patients with situs inversus before proceeding with surgery. In the present case, contrast-enhanced CT and drip-infusion cholangiography-CT were avoided due to the patient’s history of asthma, and MRCP was performed to evaluate the biliary anatomy. No vascular mapping was undertaken, and no arterial or biliary anatomical abnormalities were encountered intraoperatively.

The technical difficulties encountered during the procedure should be discussed next. The most commonly used port placement for laparoscopic cholecystectomy in patients with situs inversus is the mirrored version of the American technique, utilizing a four-port approach, which was also employed in this case [9]. In this case, during the management of the cystic artery, the hepatocystic triangle is positioned on the opposite side of the cystic duct from the port where the surgeon typically operates the energy device with the right hand. Additionally, when the Hartmann’s pouch is retracted with the left-hand forceps, the surgeon’s instruments tend to cross, further complicating the dissection process [10]. The dissection of the hepatocystic triangle is critical for confirming the CVS and ensuring the safe management of the cystic duct [11]. Dissection of the hepatocystic triangle with the right hand may contribute to prolonged operative time in patients with SIT. Some reports have suggested that left-handed surgeons may experience ergonomic advantages in this setting [9]. To address this issue, one option is for the assistant to retract the Hartmann's pouch while the right-handed surgeon dissects the hepatocystic triangle with the right hand, or to modify the port placement to improve access. Recent studies have also proposed that performing the procedure with a single-incision laparoscopic approach can improve the ease of manipulation [12].

BMI is commonly used to assess obesity. In adults, a BMI between 25.0 and 29.9 kg/m² is classified as overweight, while a BMI of 30 kg/m² or greater is classified as obese. The global prevalence of obesity has increased, and these trends have been observed in both developed and developing countries [13]. Obesity is recognized as one of the major risk factors for cholelithiasis. Therefore, the number of cholecystectomies performed in obese patients is expected to increase in the future. In general, surgical procedures in the field of gastroenterology are technically more challenging in obese patients, with reports indicating an increase in operative time and postoperative complications [14].

There have been reports that, in laparoscopic cholecystectomy, obese patients do not have higher rates of postoperative complications other than surgical site infections, but they do have a significantly increased risk of conversion to open surgery [3]. It has been reported that, when conversion to open surgery occurs, there is a significant increase in the incidence of pneumonia and surgical site infections, as well as prolonged antibiotic use and length of hospital stay, resulting in a 30% increase in healthcare costs [15]. However, according to the report by Aziz et al., patients with morbid obesity (BMI ≥ 40) have a significantly higher risk of common bile duct injury, requiring special caution. This increased risk may be attributable not only to misidentification of anatomical structures in obese patients but also to inflammatory changes and difficulty in tissue dissection [16]. In patients with situs inversus, the atypical clinical presentation may lead to delayed diagnosis of cholecystitis or biliary colic, resulting in postponed treatment [17]. The severity of inflammation may also be increased, potentially further elevating the difficulty of surgical intervention. Indeed, it has been demonstrated that patients with acute cholecystitis accompanied by preoperative inflammation have a significantly increased risk of bile duct injury. Specifically, the risk has been reported to be approximately 2.4 times higher in moderate (grade II) acute cholecystitis and nearly 8.4 times higher in severe (grade III) cases [18]. Although large-scale studies evaluating complication risk in laparoscopic cholecystectomy for patients with situs inversus and obesity are lacking, the reversed anatomy may increase technical complexity. However, in the present case, the procedure was completed safely without complications, and the patient was discharged on postoperative day 3.

Although intraoperative cholangiography has been suggested to reduce bile duct injury in patients with SIT [19], it was not performed in this case because the CVS was clearly achieved and the biliary anatomy was well defined.

## Conclusions

This case suggests that laparoscopic cholecystectomy may be feasible in patients with SIT and obesity (BMI >30 kg/m²) when careful preoperative assessment and appropriate intraoperative modifications are applied. Recognition of mirror-image anatomy and thoughtful port placement are essential to safely achieve the CVS. Our experience highlights the importance of meticulous surgical planning in managing this rare clinical scenario.
